# A Human "eFP" Browser for Generating Gene Expression Anatograms

**DOI:** 10.1371/journal.pone.0150982

**Published:** 2016-03-08

**Authors:** Rohan V. Patel, Erin T. Hamanishi, Nicholas J. Provart

**Affiliations:** 1 Department of Cell & Systems Biology, 25 Willcocks Street, University of Toronto, Toronto, ON, M5S 3B2, Canada; 2 Centre for the Analysis of Genome Evolution and Function, 25 Willcocks Street, University of Toronto, Toronto, ON, M5S 3B2, Canada; 3 Department of Biological Sciences, 1265 Military Trail, University of Toronto Scarborough, Toronto, ON, M1C 1A4, Canada; University of Jaén, SPAIN

## Abstract

Transcriptomic studies help to further our understanding of gene function. Human transcriptomic studies tend to focus on a particular subset of tissue types or a particular disease state; however, it is possible to collate into a compendium multiple studies that have been profiled using the same expression analysis platform to provide an overview of gene expression levels in many different tissues or under different conditions. In order to increase the knowledge and understanding we gain from such studies, intuitive visualization of gene expression data in such a compendium can be useful. The Human eFP (“electronic Fluorescent Pictograph”) Browser presented here is a tool for intuitive visualization of large human gene expression data sets on pictographic representations of the human body as gene expression “anatograms”. Pictographic representations for new data sets may be generated easily. The Human eFP Browser can also serve as a portal to other gene-specific information through link-outs to various online resources.

## Introduction

Global gene expression profiling studies offer an unparalleled opportunity to further our understanding of gene function. In particular, the ability to decipher when a given gene is expressed, and to what level in certain tissues and developmental stages can prove useful for human biomedical studies. It has been estimated that the human genome contains ~21,000 protein-coding genes [1], with more recent estimates putting this number even lower at ~19,000 [2]. Experimental protein-level evidence for at least 30% of the ~21,000 genes is lacking [3], leaving a sizeable void in our understanding of gene function. Gene expression profiling can help bridge this gap, by generating experimental evidence that a given gene is at least transcribed.

Expression levels of human genes vary across a multitude of tissue types, developmental stages and disease states. Typically, studies have focused on a particular subset of these conditions, but “atlas”-type resources such as the Genomics Institute of the Novartis Research Foundation (GNF) Gene Expression Atlas (Su et al., 2004) that encompasses a wide variety of tissue types and disease states have also been generated. Integration of a number of independent microarray studies covering a wide variety of biological conditions is challenging but possible as long as they have been sampled using the same platform [4]. We have integrated several such studies found both in the Gene Expression Omnibus (GEO, [5]) and ArrayExpress [6]. This includes samples from the GNF Gene Expression Atlas as well as the following series: GSE475, GSE2361 [7], GSE3526 [8], GSE8961 [9], GSE4567 [10], GSE7307 [11], GSE19650 [12], E-MTAB-47 [13], E-GEOD-6257 [14], and E-MEXP-2219 [15]. In total, 774 samples from 11 different data sets have been collated. In addition to this, the RNA-Seq Illumina Human BodyMap 2.0 data set ([16]; Ensembl Release 70) containing 16 different samples has been added to the Human eFP Browser, showing the flexibility of this tool to enable viewing of data from different platforms (expression levels for a given gene and tissue combination are not directly comparable if generated by different platforms—a message at the top of the Illumina Body Map 2 view alerts users to this fact).

Ultimately, in order to maximize the potential that gene expression studies offer, the ability to rapidly and easily interrogate these data sets is necessary. The interpretation of the gene expression level values should also occur in a coherent and user-friendly manner. Many online resources exist that enable a user to visualize gene expression levels in a data set for a given gene. Such tools include BioGPS [17], EBI Expression Atlas [18], GeneCards [19], Human Protein Atlas [20], GEO Profiles [5], TiGER [21], and Genevestigator [22]. However, these tools don’t provide biological context: outputs are bar graph or heatmap visualizations, with the name of the sample being the only, often somewhat cryptic, indication as to what kind of tissue or cell type that sample was generated from. A more informative way to visualize such data would be to show the level of expression in an anatomical sense, thus lending some context to the data. While the Expression Atlas tool at the EBI [23] does provide a representation of the human body for the Illumina Human Body Map 2.0 data set [16], where the corresponding body part is highlighted if a user moves his/her mouse over the gene expression value of interest, eye saccades and top-down processes [24] are required to actually determine to which part of the body a given expression value belongs. This user interface also fails to provide anatomical context for smaller structures within tissues.

Here, we present a tool that enables the user to visualize large-scale human gene expression data sets directly on representations of the human body—the Human eFP Browser at http://bar.utoronto.ca/efp_human/, which is based on an open source framework developed by Winter et al. (2007). Current data sets in the Human eFP Browser were sampled on the HG-U133A and HG-U133 Plus 2 arrays (Affymetrix Inc., Santa Clara, USA), and by RNA-seq in the case of the Illumina Body Map 2 view. The user is shown diagrammatic anatomical representations that correspond to those areas of the body that were used to generate the RNA samples described above (currently categorized into five different views). The normalized gene expression data are stored on the Bio-Analytic Resource (BAR) server [25]. The user enters an Entrez gene identifier, a gene symbol, or a probe set identifier, and then chooses the mode of interpretation (absolute, relative, or compare). After clicking “Go”, the representations of human samples are coloured based on the expression level of the gene of interest, generating expression “anatograms” for rapidly determining where a given gene is most strongly expressed. A yellow-red scale is used in the “Absolute” mode to depict expression levels, with yellow denoting no expression in a given depiction of a tissue and red denoting maximal expression. “Relative” mode displays the ratio of the expression level of a given gene relative to a control level (the median expression level for that gene across all samples in a particular view). The colour scale used in this instance is yellow-red for values above the control level, and yellow-blue for values below the control level. In “Compare” mode the primary gene expression level is compared to that of the secondary gene expression level, and the colour scheme is the same as in the “Relative” mode.

Information regarding the view with the highest level of gene expression is given near the top of the view, and information regarding probe set/gene identifiers as well as functional annotation attributed to the query gene is given at the bottom. Since gene expression data are given anatomical context, further interpretation is allowed and data become more accessible to users who may not be completely familiar with all parts of human anatomy. The Human eFP Browser is intended as a rapid and easy means for visualizing gene expression data sets to identify gene expression patterns of interest and facilitate hypothesis generation. Gene-specific link-outs are also provided to corresponding gene records in BioGPS [17], the Gene database at NCBI [26], UniProt [27], EBI, and GeneMANIA [28]. Thus the Human eFP Browser can also serve as a portal to gene-specific information. We have also worked with the curators at NCBI such that link-outs to the Human eFP Browser are available from the human Gene pages at NCBI.

## Results

**Fig 1:**
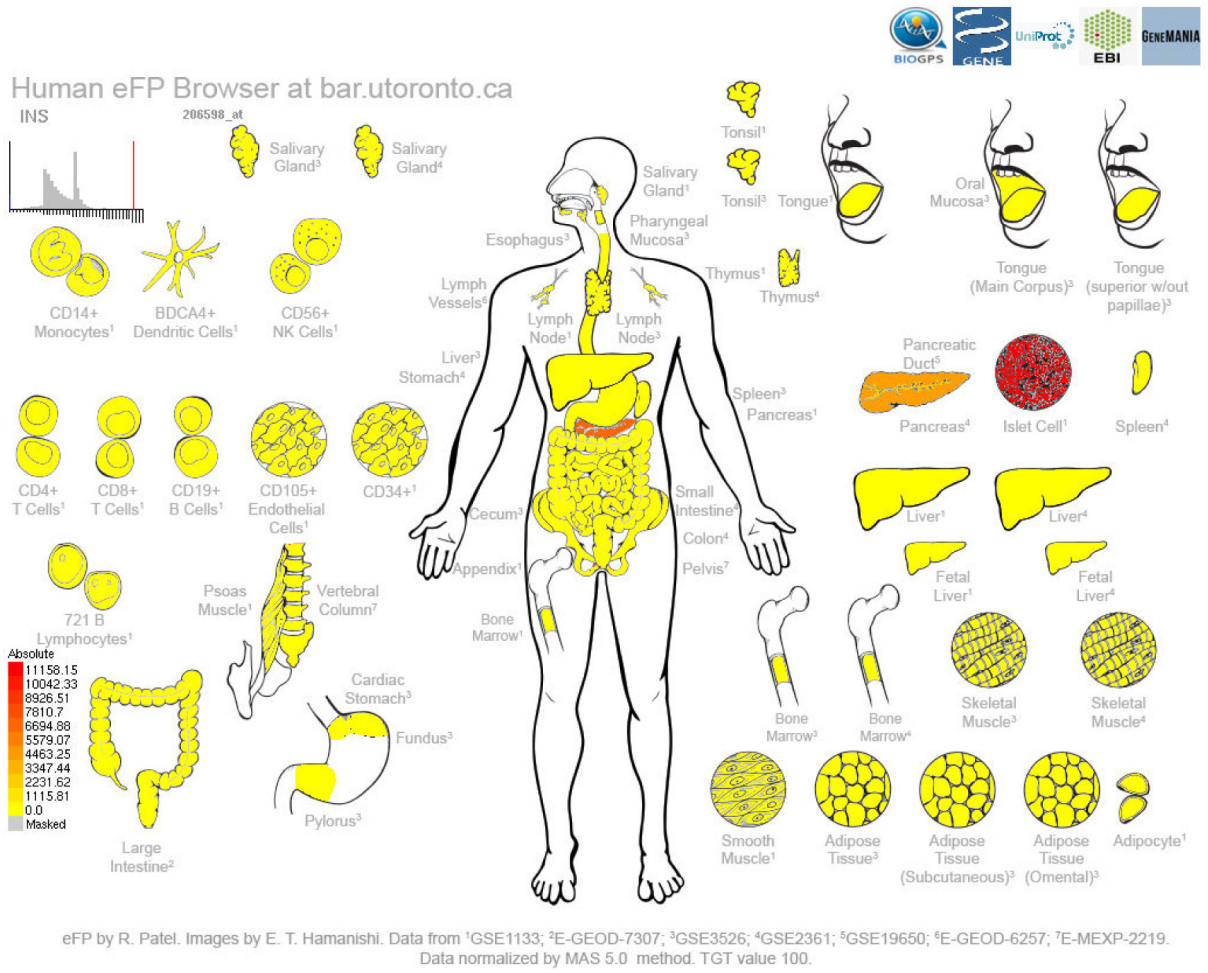
Human eFP Browser output showing expression patterns of the *INS* gene in the “Skeletal Immune Digestive” compendium. Strong expression of *INS*, as denoted by the red colour, is observed in Islet cell cultures, and to a lesser extent in RNA samples generated from the whole pancreas, where these specialized cells are found.

In order to demonstrate the utility of the Human eFP Browser, we present examples of genes whose expression patterns have been published. The first example output shown in Fig 1 is for the insulin (*INS*) gene, which is expressed most highly in the pancreatic β islet cells [29]. Here, the gene symbol (“INS”) was entered, “Absolute” mode was selected and the “Skeletal Immune Digestive” data source was also selected. The output for this gene shows expression exclusively in the pancreas / islet cells. Also any functional annotation attributed to the gene is given (not shown). Direct links to the records for the *INS* gene in BioGPS, NCBI, UniProt, and EBI are provided at the top of the output.

**Fig 2:**
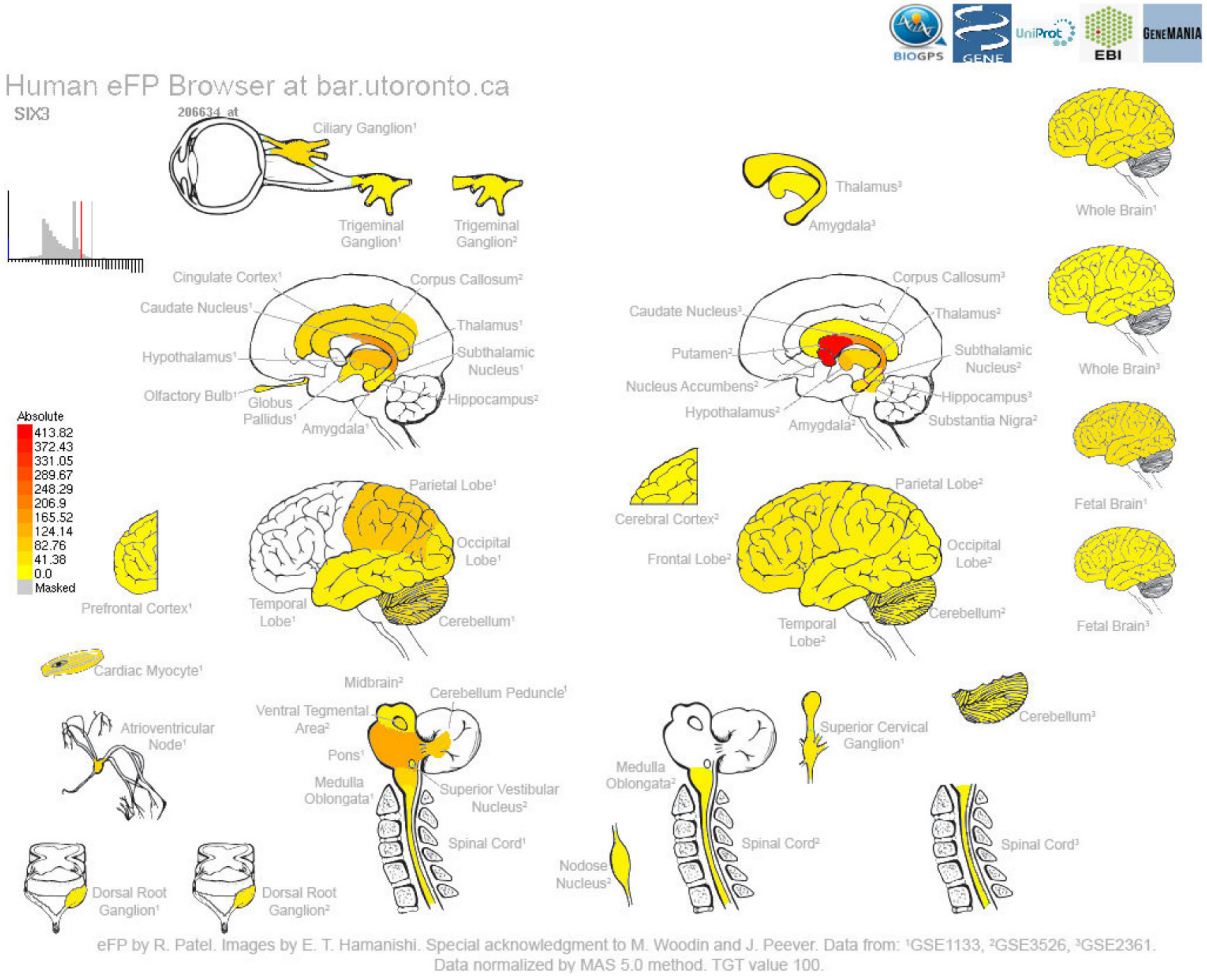
Human eFP Browser output for the *SIX homeobox 3* (*SIX3*) gene using the “Nervous” Data Source, showing strong levels of expression in the putamen and nucleus accumbens, as denoted by the red colouring.

A second example output is shown in Fig 2 and is for the *SIX homeobox 3* (*SIX3*) gene, which is associated with developmental abnormalities in the forebrain [30]. The highest levels of gene expression are found in the putamen and nucleus accumbens. Again, additional information related to this gene as well as link-outs to other resources are provided.

**Fig 3:**
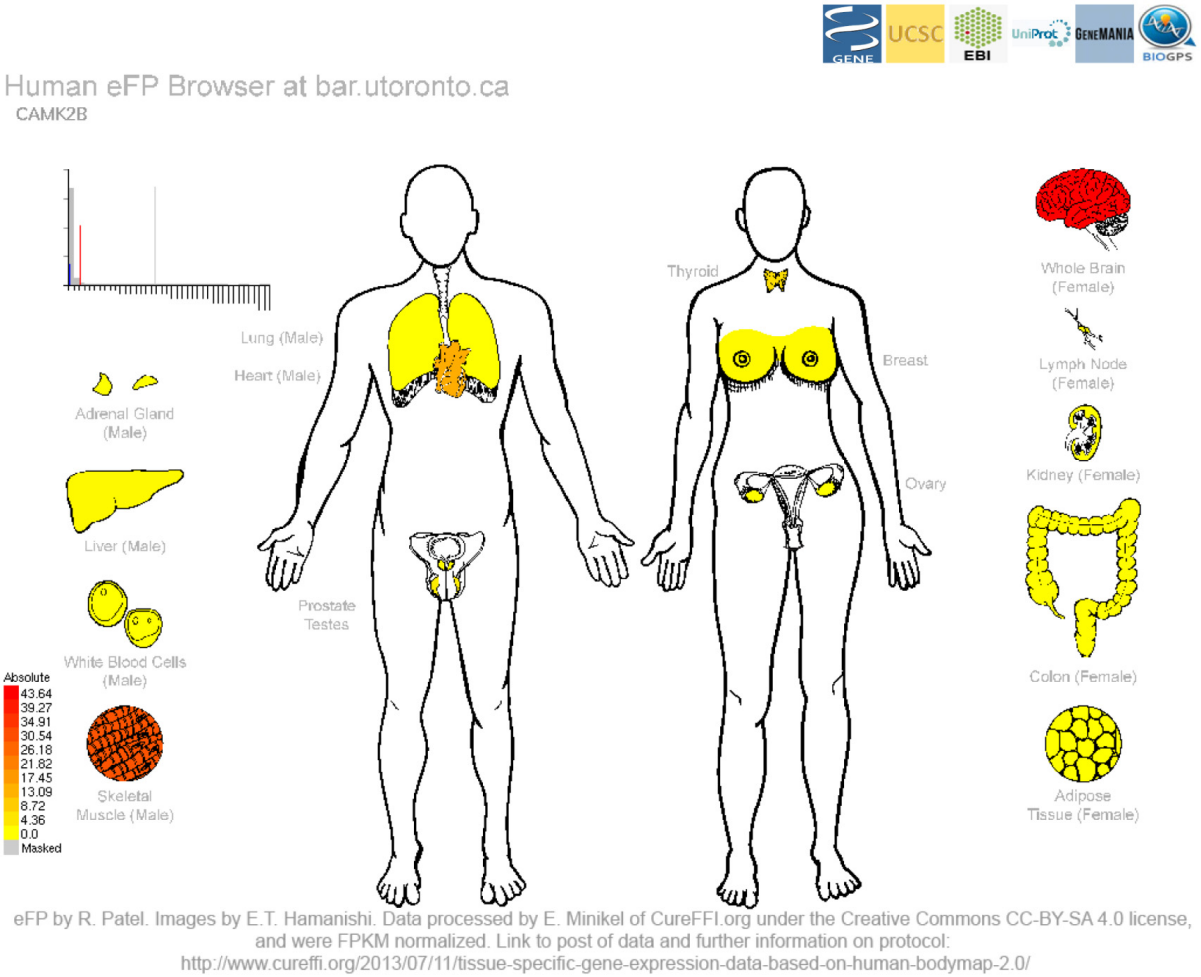
Human eFP Browser output for the *calcium/calmodulin-dependant protein kinase II beta* (*CAMK2B*) gene using the “Human Body Map 2 Illumina” Data Source. Highest expression levels are found in the brain and to a lesser extent in the skeletal muscle, as denoted by the red colouring.

The *calcium/calmodulin-dependant protein kinase II beta* (*CAMK2B*) gene is the final output example and its expression patterns are shown in Fig 3. It is involved in neuronal plasticity and synapse formation [31]. In the RNA-Seq Human eFP Browser view, highest expression levels are found in the brain and to a lesser extent in the skeletal muscle. In this view, it is also possible to view related information and link outs to other resources.

## Discussion

When considering global microarray or RNA-seq gene expression profiling studies, gene expression levels are a useful guide to that gene’s biology. The Human eFP Browser provides users with the ability to easily visualize and rapidly interpret the results of gene expression studies in humans. While many human gene expression studies focus on a particular area of the human body, this tool enables the user to interpret gene expression levels across multiple tissue types. Moreover, for users who are less familiar with human anatomy, such expression data sets will become more accessible as the data are given anatomical context, as opposed to being shown as a bar graph.

In order to provide examples of the utility of the Human eFP Browser, we chose three genes that are expected to show high levels of gene expression in specific tissues. *INS* shows highest expression in the islet cells (Fig 1), while *SIX3* shows highest expression in the putamen and nucleus accumbens (Fig 2), and *CAMK2B* shows highest expression in the brain (Fig 3). These examples show the utility of this tool for visualizing gene expression data sets (both microarray- and RNA-seq-based).

At present, link-outs are provided several common repositories for gene information in order to provide further details at the click of a mouse. Users can also access the relevant experiment records in GEO by clicking on individual tissues on the image. Additionally, on mouse-over the tissue name and expression value (absolute, or relative with fold-change or standard deviation) is displayed. Underneath the main image, a link is provided to a table listing all sample names, expression values, fold-changes, and standard deviations, as well as a chart showing the same information. Gene specific link-outs to entries in other databases can be found above the main image.

In the future, as more human gene expression experiments are conducted, we envisage adding further data sets and views to this tool, including those that have been profiled on other platforms. Current and future activities involve adding further developmental data sets, as well as disease data sets e.g. cancer gene expression studies, into the Human eFP Browser. In this way, the Human eFP Browser can become a comprehensive resource for visualization and interpretation of human gene expression data and an aggregator of link-outs to various other resources. We encourage any researcher to contact us with ideas for specific views.

## Materials and Methods

A number of human microarray data sets are represented within the Human eFP Browser. From GEO, the following data sets are represented: GSE1133, GSE475, GSE2361, GSE3526, GSE8961, GSE4567, GSE7307, and GSE19650. Other data sets are from ArrayExpress: E-MTAB-47, E-GEOD-6257, and E-MEXP-2219. All microarray data sets were normalized in R/Bioconductor using the MAS 5 method with a target value of 100 with the following commands:

#Load affy package
> library(affy)
#Set working directory to directory containing the data you wish to normalize
> setwd("[FULL PATH TO DIRECTORY CONTAINING THE DATA]")
#Invoke ReadAffy to define specific cdf
> GSE35261<-ReadAffy(cdfname = "hgu133acdf")
#MAS 5 normalize the data with a tgt value of 100, and the defined cdf file
> GSE35261Norm<-mas5(GSE35261, sc = 100)
#Write the data to a csv file
> write.exprs(GSE35261Norm, file = "GSE35261Norm_tgt100.csv")

The RNA-Seq FPKM processed data set was processed by Eric Minikel of cureFFI.org (http://www.cureffi.org/2013/07/11/tissue-specific-gene-expression-data-based-on-human-bodymap-2-0/). The processing by Eric Minikel prior to our download was as follows: Ensembl BAM files were downloaded. Cufflinks was used to summarize expression levels as FPKM values. Only known transcripts were called.

The Human eFP Browser is implemented in Python, and inputs include a Targa-based image, XML control file, gene identifier to microarray probe set lookup and annotation databases, and a gene expression database for the given samples. These components work together to produce an output image, as described in Winter et al. (2007). The eFP Browser open source code is available at http://sourceforge.net/projects/efpbrowser/ and original expression data may be downloaded from GEO or ArrayExpress using the accession numbers on the previous page. Processed data are at https://github.com/asherpasha/eFP_Human_Databases under the DOI of 10.5281/zenodo.45940.

## References

[1] Clamp M, Fry B, Kamal M, Xie X, Cuff J, Lin MF, et al Distinguishing protein-coding and noncoding genes in the human genome. Proc Natl Acad Sci. 2007;104: 19428–19433. 10.1073/pnas.0709013104 18040051 PMC2148306

[2] Ezkurdia I, Juan D, Rodriguez JM, Frankish A, Deikhans M, Harrow JL, et al. The shrinking human protein coding complement: are there fewer than 20,000 genes? [Internet]. 2014 Jan. Report No.: 001909. Available: http://biorxiv.org/lookup/doi/10.1101/001909.

[3] Legrain P, Aebersold R, Archakov A, Bairoch A, Bala K, Beretta L, et al The human proteome project: current state and future direction. Mol Cell Proteomics MCP. 2011;10: M111.009993 10.1074/mcp.M111.009993 21742803 PMC3134076

[4] Lukk M, Kapushesky M, Nikkilä J, Parkinson H, Goncalves A, Huber W, et al A global map of human gene expression. Nat Biotechnol. 2010;28: 322–324. 10.1038/nbt0410-322 20379172 PMC2974261

[5] Barrett T, Wilhite SE, Ledoux P, Evangelista C, Kim IF, Tomashevsky M, et al NCBI GEO: archive for functional genomics data sets—update. Nucleic Acids Res. 2013;41: D991–D995. 10.1093/nar/gks1193 23193258 PMC3531084

[6] Rustici G, Kolesnikov N, Brandizi M, Burdett T, Dylag M, Emam I, et al ArrayExpress update—trends in database growth and links to data analysis tools. Nucleic Acids Res. 2013;41: D987–D990. 10.1093/nar/gks1174 23193272 PMC3531147

[7] Ge X, Yamamoto S, Tsutsumi S, Midorikawa Y, Ihara S, Wang SM, et al Interpreting expression profiles of cancers by genome-wide survey of breadth of expression in normal tissues. Genomics. 2005;86: 127–141. 10.1016/j.ygeno.2005.04.008 15950434

[8] Roth RB, Hevezi P, Lee J, Willhite D, Lechner SM, Foster AC, et al Gene expression analyses reveal molecular relationships among 20 regions of the human CNS. Neurogenetics. 2006;7: 67–80. 10.1007/s10048-006-0032-6 16572319

[9] Bao X, Sinha M, Liu T, Hong C, Luxon BA, Garofalo RP, et al Identification of human metapneumovirus-induced gene networks in airway epithelial cells by microarray analysis. Virology. 2008;374: 114–127. 10.1016/j.virol.2007.12.024 18234263 PMC2777699

[10] Karoly ED, Li Z, Dailey LA, Hyseni X, Huang Y-CT. Up-regulation of tissue factor in human pulmonary artery endothelial cells after ultrafine particle exposure. Environ Health Perspect. 2007;115: 535–540. 17450221 10.1289/ehp.9556 PMC1852686

[11] Richard B Roth. Available: http://www.ncbi.nlm.nih.gov/geo/query/acc.cgi?acc=GSE7307.

[12] Hiraoka N, Yamazaki-Itoh R, Ino Y, Mizuguchi Y, Yamada T, Hirohashi S, et al CXCL17 and ICAM2 are associated with a potential anti-tumor immune response in early intraepithelial stages of human pancreatic carcinogenesis. Gastroenterology. 2011;140: 310–321. 10.1053/j.gastro.2010.10.009 20955708

[13] Røe OD, Anderssen E, Helge E, Pettersen CH, Olsen KS, Sandeck H, et al Genome-wide profile of pleural mesothelioma versus parietal and visceral pleura: the emerging gene portrait of the mesothelioma phenotype. PloS One. 2009;4: e6554 10.1371/journal.pone.0006554 19662092 PMC2717215

[14] Johnson LA, Clasper S, Holt AP, Lalor PF, Baban D, Jackson DG. An inflammation-induced mechanism for leukocyte transmigration across lymphatic vessel endothelium. J Exp Med. 2006;203: 2763–2777. 10.1084/jem.20051759 17116732 PMC2118156

[15] Kalogeropoulos M, Varanasi SS, Olstad OK, Sanderson P, Gautvik VT, Reppe S, et al Zic1 transcription factor in bone: neural developmental protein regulates mechanotransduction in osteocytes. FASEB J Off Publ Fed Am Soc Exp Biol. 2010;24: 2893–2903. 10.1096/fj.09-148908 20354137

[16] Flicek P, Ahmed I, Amode MR, Barrell D, Beal K, Brent S, et al Ensembl 2013. Nucleic Acids Res. 2013;41: D48–55. 10.1093/nar/gks1236 23203987 PMC3531136

[17] Wu C, MacLeod I, Su AI. BioGPS and MyGene.info: organizing online, gene-centric information. Nucleic Acids Res. 2013;41: D561–D565. 10.1093/nar/gks1114 23175613 PMC3531157

[18] Petryszak R, Burdett T, Fiorelli B, Fonseca NA, Gonzalez-Porta M, Hastings E, et al Expression Atlas update—a database of gene and transcript expression from microarray- and sequencing-based functional genomics experiments. Nucleic Acids Res. 2014;42: D926–932. 10.1093/nar/gkt1270 24304889 PMC3964963

[19] Safran M, Dalah I, Alexander J, Rosen N, Iny Stein T, Shmoish M, et al GeneCards Version 3: the human gene integrator. Database J Biol Databases Curation. 2010;2010: baq020 10.1093/database/baq020 PMC2938269 20689021

[20] Uhlen M, Oksvold P, Fagerberg L, Lundberg E, Jonasson K, Forsberg M, et al Towards a knowledge-based Human Protein Atlas. Nat Biotechnol. 2010;28: 1248–1250. 10.1038/nbt1210-1248 21139605

[21] Liu X, Yu X, Zack DJ, Zhu H, Qian J. TiGER: a database for tissue-specific gene expression and regulation. BMC Bioinformatics. 2008;9: 271 10.1186/1471-2105-9-271 18541026 PMC2438328

[22] Hruz T, Laule O, Szabo G, Wessendorp F, Bleuler S, Oertle L, et al Genevestigator v3: a reference expression database for the meta-analysis of transcriptomes. Adv Bioinforma. 2008;2008: 420747. 10.1155/2008/420747 PMC2777001 19956698

[23] Expression Atlas. Available: https://www.ebi.ac.uk/gxa/experiments/E-MTAB-513.

[24] Ware C. Information Visualization: Perception for Design. Elsevier; 2012.

[25] Toufighi K, Brady SM, Austin R, Ly E, Provart NJ. The Botany Array Resource: e-Northerns, Expression Angling, and promoter analyses. Plant J. 2005;43: 153–163. 10.1111/j.1365-313X.2005.02437.x 15960624

[26] Maglott D, Ostell J, Pruitt KD, Tatusova T. Entrez Gene: gene-centered information at NCBI. Nucleic Acids Res. 2011;39: D52–D57. 10.1093/nar/gkq1237 21115458 PMC3013746

[27] Activities at the Universal Protein Resource (UniProt). Nucleic Acids Res. 2014;42: D191–D198. 10.1093/nar/gkt1140 24253303 PMC3965022

[28] Warde-Farley D, Donaldson SL, Comes O, Zuberi K, Badrawi R, Chao P, et al The GeneMANIA prediction server: biological network integration for gene prioritization and predicting gene function. Nucleic Acids Res. 2010;38: W214–220. 10.1093/nar/gkq537 20576703 PMC2896186

[29] Mutskov V, Felsenfeld G. The human insulin gene is part of a large open chromatin domain specific for human islets. Proc Natl Acad Sci. 2009;106: 17419–17424. 10.1073/pnas.0909288106 19805079 PMC2765094

[30] Miller JA, Ding S-L, Sunkin SM, Smith KA, Ng L, Szafer A, et al Transcriptional landscape of the prenatal human brain. Nature. 2014;508: 199–206. 10.1038/nature13185 24695229 PMC4105188

[31] Okamoto K, Bosch M, Hayashi Y. The Roles of CaMKII and F-Actin in the Structural Plasticity of Dendritic Spines: A Potential Molecular Identity of a Synaptic Tag? Physiology. 2009;24: 357–366. 10.1152/physiol.00029.2009 19996366

